# Mobile-based oral cancer classification for point-of-care screening

**DOI:** 10.1117/1.JBO.26.6.065003

**Published:** 2021-06-23

**Authors:** Bofan Song, Sumsum Sunny, Shaobai Li, Keerthi Gurushanth, Pramila Mendonca, Nirza Mukhia, Sanjana Patrick, Shubha Gurudath, Subhashini Raghavan, Tsusennaro Imchen, Shirley T Leivon, Trupti Kolur, Vivek Shetty, Vidya Bushan, Rohan Ramesh, Natzem Lima, Vijay Pillai, Petra Wilder-Smith, Alben Sigamani, Amritha Suresh, Moni A. Kuriakose, Praveen Birur, Rongguang Liang

**Affiliations:** a The University of Arizona, Wyant College of Optical Sciences, Tucson, Arizona, United States; b Mazumdar Shaw Medical Centre, Bangalore, Karnataka, India; c KLE Society’s Institute of Dental Sciences, Bangalore, Karnataka, India; d Mazumdar Shaw Medical Foundation, Bangalore, Karnataka, India; e Biocon Foundation, Bangalore, Karnataka, India; f Christian Institute of Health Sciences and Research, Dimapur, Nagaland, India; g University of California, Beckman Laser Institute and Medical Clinic, Irvine, California, United States; h Cochin Cancer Research Center, Kochi, Kerala, India

**Keywords:** oral cancer, mobile screening device, dual-modality, efficient deep learning

## Abstract

**Significance:** Oral cancer is among the most common cancers globally, especially in low- and middle-income countries. Early detection is the most effective way to reduce the mortality rate. Deep learning-based cancer image classification models usually need to be hosted on a computing server. However, internet connection is unreliable for screening in low-resource settings.

**Aim:** To develop a mobile-based dual-mode image classification method and customized Android application for point-of-care oral cancer detection.

**Approach:** The dataset used in our study was captured among 5025 patients with our customized dual-modality mobile oral screening devices. We trained an efficient network MobileNet with focal loss and converted the model into TensorFlow Lite format. The finalized lite format model is ∼16.3  MB and ideal for smartphone platform operation. We have developed an Android smartphone application in an easy-to-use format that implements the mobile-based dual-modality image classification approach to distinguish oral potentially malignant and malignant images from normal/benign images.

**Results:** We investigated the accuracy and running speed on a cost-effective smartphone computing platform. It takes ∼300  ms to process one image pair with the Moto G5 Android smartphone. We tested the proposed method on a standalone dataset and achieved 81% accuracy for distinguishing normal/benign lesions from clinically suspicious lesions, using a gold standard of clinical impression based on the review of images by oral specialists.

**Conclusions:** Our study demonstrates the effectiveness of a mobile-based approach for oral cancer screening in low-resource settings.

## 1 Introduction

Oral cancer is a highly prevalent cancer; 377,713 new cases and 177,757 deaths are reported each year worldwide.^1^ India alone accounts for more than 33% (135,929) of new cases and 20% (75,290) of deaths, according to Globocan 2020.^1^ The second most common cancer in India is oral cancer,^2^ and its pervasiveness is increasing rapidly. From 2018 to 2020, the number of new cases increased by 12% (from 119,992 to 135,929) according to comparisons from Globocan 2018 and Globocan 2020.^1^^,^^3^ According to the World Health Organization, the estimated number of new cases and deaths in Asia will increase to 374,000 and 208,000 by 2040.^4^ It is a growing problem and a leading cause of mortality in specific regions such as India.^5^^,^^6^ Detecting oral cancer at an early stage is believed to be the most effective way of (1) reducing the individual burden, (2) decreasing morbidity and mortality, and (3) improving quality of life.^7^ Most high-risk individuals live in resource-limited settings that lack trained specialists and health services.^8^^,^^9^ Conventional oral examination and biopsy, the clinical and gold standard for oral lesion detection, are not ideal as screening tools in these regions.^10^ Therefore, the chances of survival are reduced due to delayed diagnosis.^11^

Convolutional neural networks (CNNs)^12^ are a powerful tool to solve image classification problems^13^ and have been widely used for many medical image analysis tasks with great performance.^14^^,^^15^ Deep CNNs show potential for automated classification of different types of cancer lesions, such as skin cancer,^16^ cervical cancer,^17^^,^^18^ and oral cancer.^19^^,^^20^ A smartphone-based, point-of-care platform outfitted with automated classification powered by deep learning techniques can extend the reach of trained specialists outside of the clinic to provide low-cost universal access to vital diagnostic care. Deep learning models suffer from being large, complicated, slow, and typically need high-performance computing to run. Mobile platforms are small and inexpensive; however, they do not have high-performance computing capabilities. One common solution is to host an AI cloud server, upload the image to the cloud server for processing, and then download the results.^21^ For this approach, internet connection is the bottleneck. It is not easy to get internet access everywhere, especially for resource-limited settings, much less at high-speed upload and download data rates. Rural India only has 18% mobile internet penetration, according to a report by the Internet and Mobile Association of India.^22^

This paper describes the development of an automated, dual-modality, oral potentially malignant lesion (OPML) and malignant lesion image classification system using the deep learning model MobileNet.^23^ The platform can run quickly with high accuracy in resource-constrained settings where computation power and internet access is limited. The deep learning-based classification and image preprocessing user interface can be accessed by clinicians through an easy-to-use Android smartphone application for real-time classification results of patients in resource-limited settings, regardless of internet connection. The network is trained to identify clinically suspicious lesions based on a gold standard of clinical impression. Experimental results with the developed application confirm the effectiveness and efficiency of the automated, dual-modality, oral potentially, and malignant image classification system approach on mobile devices for identifying clinically suspicious lesions.

## 2 Methods

**Fig. 1:**
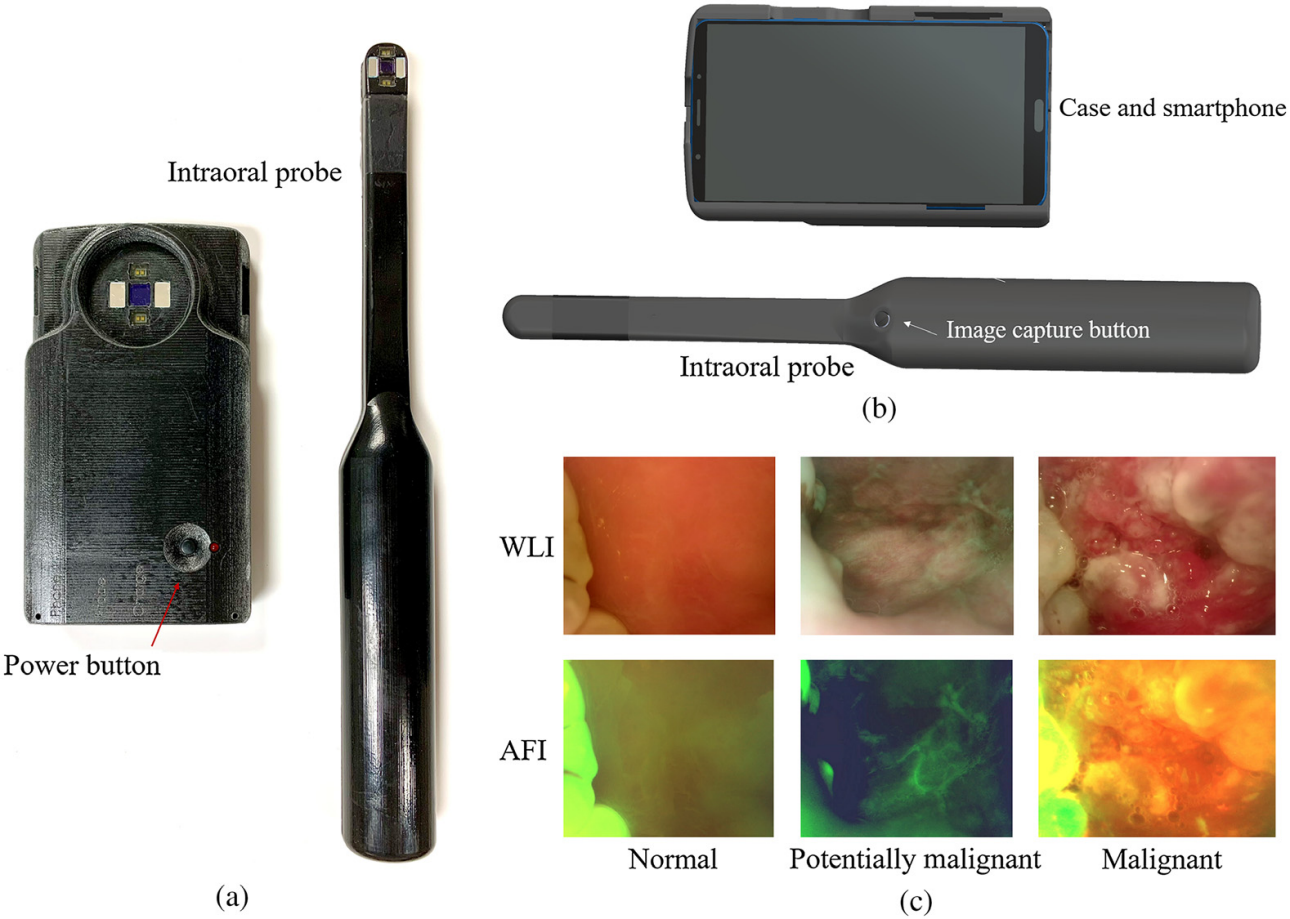
The (a) back and (b) front side: the dual-modal oral cancer screening system consists of a Moto G5 smartphone, a phone case with battery and electronics, an intraoral probe, an OTG cable connection from the phone case to the smartphone, and a cable connection from the intraoral probe to the phone case. The head of the intraoral probe has white-light LEDs, violet LEDs, and a camera module. Narrowband filters and a long-pass filter are placed in front of the violet LEDs and camera to enable intraoral autofluorescence imaging. The polarizing filter has been put in front of the white-light LEDs and camera to suppress glare from the saliva on mucosa surface. A custom Android application controls the system, captures dual-modal images, and implements the MobileNet based on-device dual-modal classification system in an easy-to-use format. (c) The three example image pairs of the WLI and AFI are shown in the figure labeled “normal,” “potentially malignant,” and “malignant” by oral specialists. The image pair labeled normal would be categorized as negative by the gold standard (normal/benign). The image pairs labeled potentially malignant and malignant would both be categorized as positive by the gold standard [suspicious (OPMD/malignant)]. The algorithm is trained to classify normal/benign versus suspicious.

Figure 1 shows our smartphone-based, dual-modal oral cancer screening platform that runs our deep learning-based approach.^24^^,^^25^ The platform consists of a commercial Moto G5 Android smartphone, an intraoral imaging probe, an light-emitting diode (LED) driver, a rechargeable lithium battery, and mobile application. The system utilizes four 405-nm Luxeon UV U1 LEDs (Lumileds, Amsterdam, Netherlands) to enable intraoral autofluorescence imaging (AFI) and four 4000-K Luxeon Z ES LEDs for intraoral white-light imaging (WLI). The illumination LEDs are driven by a switching boost voltage regulator (LT1815, Linear Technology, Milpitas, CA). The custom Android application provides the user interface, controls the phone and probe, captures dual-modal intraoral images, and implements our oral image classification approach.

For visual oral cancer diagnosis, AFI can help detect some OPMLs and malignant lesions not visible by WLI and vice versa.^26^^–^^28^ Previous studies conclude that better accuracy could be achieved by combining AFI and WLI.^29^ Dual-modal images using AFI and WLI have proven to improve accuracy in oral cancer image classification, as shown in our previous study.^24^ Dual-modality images fuse information from AFI and WLI to feed the CNN. For AFI, a red channel to green channel ratio map is created because it is an important feature correlated with OPMLs (oral premalignant lesions) and malignant lesions. For WLI, the blue channel is excluded because the long-pass filter blocks light beneath the 470-nm wavelength. The normalized ratio map from AFI and two remaining channels from WLI were combined to feed the MobileNet neural network.

**Fig. 2:**
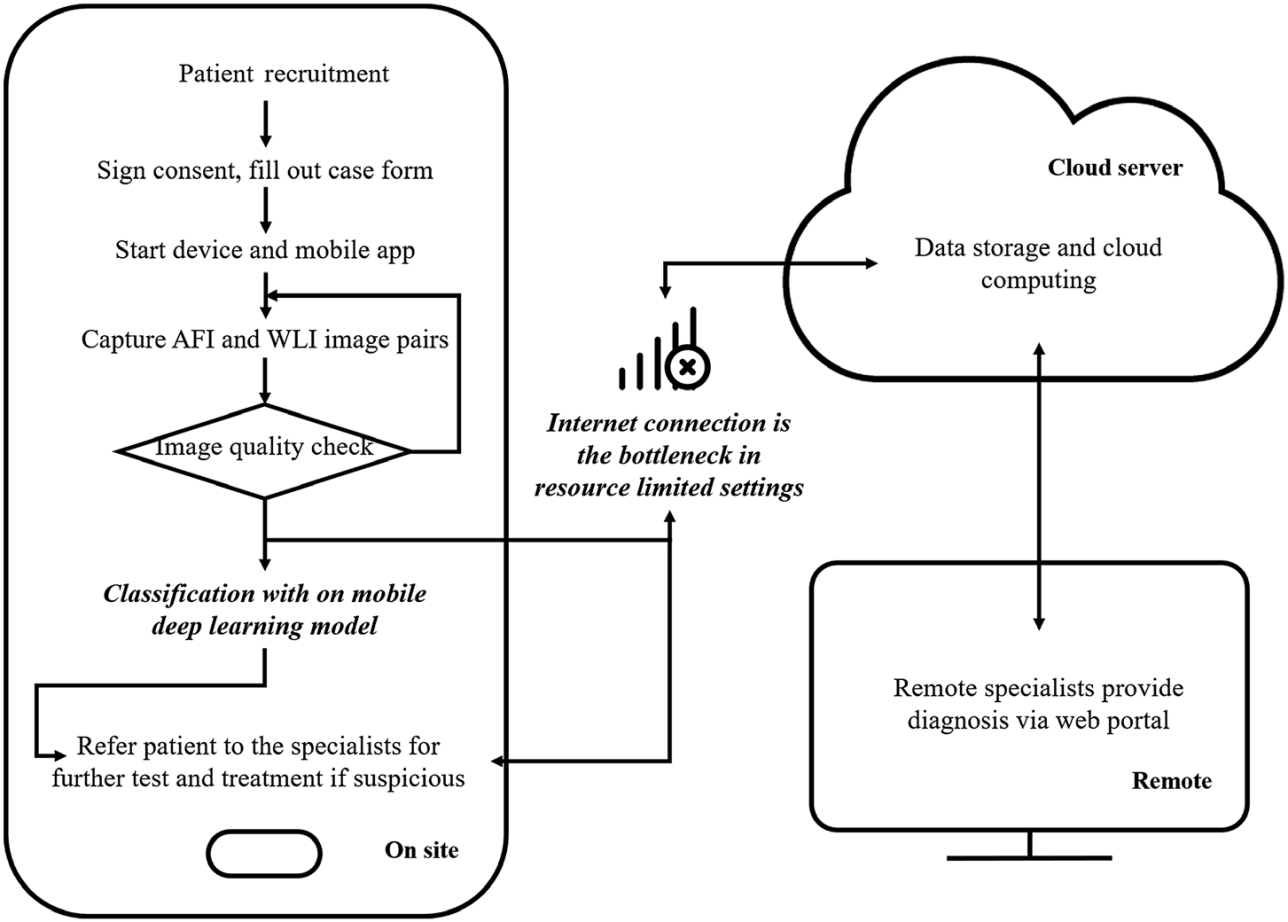
Workflow of our mobile-based oral cancer screening and classification system. The images captured from smartphone could be classified directly on the device in resource-limited setting with poor internet connection.

The workflow of the proposed mobile-based oral cancer classification system is shown in Fig. 2. The autofluorescence and WLI pairs are captured on-site and analyzed directly on the smartphone device if the internet connection is poor, such as in a resource-limited setting. The data will then be uploaded to a cloud server when internet connection is available. The deep learning model deployed on the cloud server will also classify the data. Next, remote specialists can log into the platform and provide diagnosis via a web browser.

**Fig. 3:**
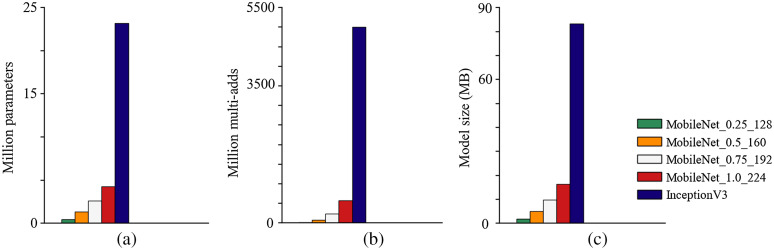
Comparison of (a) parameters, (b) operations (Multi-Adds), and (c) model sizes of MobileNet (different hyperparameters settings) with InceptionV3.

MobileNet operates by converting the standard convolutional layers to a more efficient format, depth-wise separable convolution.^30^ Depth-wise separable convolution is small, has low latency, and has low power consumption, characteristics that meet the needs of real-time, high-accuracy analysis for on-device embedded applications. The depth-wise separable convolution layer is made of a depth-wise convolution and a pointwise convolution. The depth-wise convolution layer filters each of the input channels, and the pointwise convolution layer combines the results through the depth-wise convolution layer. The computational cost and model size are drastically reduced because of the conversion. The computational cost of standard convolution is $D_{f}\times D_{f}\times M\times N\times D_{k}\times D_{k}$, where $D_{f}$ is the spatial width and height of the input feature map, M is the number of input channels, $D_{k}$ is the spatial dimension of the kernel, and N is the number of output channels. However, the computational cost of the depth-wise separable convolution is $D_{f}\times D_{f}\times M\times D_{k}\times D_{k}+D_{f}\times D_{f}\times M\times N$. By converting standard convolution to the depthwise separable convolution, we get a reduction in the computation of $(D_{f}\times D_{f}\times M\times D_{k}\times D_{k}+D_{f}\times D_{f}\times M\times N)=1/N+1/D_{k}^{2}$. Since the aim of lightweight efficient neural networks is to achieve the highest accuracy with the fastest speed, which is a contradiction in nature, MobileNet introduces two hyperparameters to trade-off between speed and accuracy. First, the width multiplier reduces the number of input/output channels of each layer. Second, the resolution multiplier reduces the resolution of every layer input. The InceptionV3 network was used for comparison. InceptionV3, based on inception deep convolutional architecture with additional factorization ideas, has been widely used for many applications. Figure 3 shows the comparison of operations, parameters, and model sizes of MobileNet with InceptionV3. The operations were quantified using Multi-Adds, which represents the number of multiply-add operations of a deep learning model, smaller Multi-Adds means the model needs fewer operations and leads to lower computational costs. MobileNet with depth-wise separable convolutions tremendously reduces the computational cost, number of parameters, and model size with only small accuracy compromises.

The performance of the proposed MobileNet-based dual-modality approach for classification of normal/benign versus suspicious lesions based on a gold standard of clinical impression was validated using the image dataset collected in India with the dual-modality mobile oral screening devices. The dataset was captured among 5025 patients attending the outpatient clinics of the Department of Oral Medicine and Radiology at KLE Society Institute of Dental Sciences, Head and Neck Oncology Department of Mazumdar Shaw Medical Center (MSMC), and Christian Institute of Health Sciences and Research (CIHSR), India. Institutional ethical committee approval was obtained from all participating hospitals and written informed consent was collected from all subjects enrolled. Oral oncology specialists from MSMC, KLE, and CIHSR labeled all the dual-modality image pairs and separated them into two categories: (A) “normal” which contains normal and benign mucosal lesion images and (B) “suspicious” which contains OPML and malignant lesion images. In a previous study, we showed that oral oncology specialists’ interpretation of classifying normal/benign versus OPML/malignant has high accuracy with biopsy-confirmed cases.^31^

We collected a total of 7970 pair of intraoral images from 5025 patients. Undiagnosable and low image quality images such as “unclear” or “not well focused” were excluded from the dataset. After exclusion, only 6211 image pairs remained. We used 5329 image pairs for training and validation. Through random assignment, 75% of the image pairs were allocated to training and 25% of the image pairs were allocated to validation. For the standalone test, we used 882 image pairs that were stratified at the patient level. Tables 1 and 2 show the lesion type distribution of the training/validation dataset and the standalone test dataset. Table 3 shows the lesion site distribution of the standalone test dataset.

**Table 1:** The lesion type distribution of the training/validation dataset.

Lesion types	Number of image pairs
Normal	3211
Benign	740
OPML	1346
Malignancy	32

**Table 2:** The lesion type distribution of the standalone test dataset.

Lesion types	Number of image pairs
Normal	393
Benign	69
OPML	397
Malignancy	23

**Table 3:** The lesion site distribution of the standalone test dataset.

Lesion sites	Number of image pairs
Cheek mucosa	604
Lower vestibule	42
Tongue	46
Lip	130
Floor of mouth	3
Palate	10
RMT	29
Other	18

We trained the network with the focal loss and the RMSProp optimization algorithm implemented on Tensorflow. The batch size for each iteration was 32, the learning rate was 0.0001, and the epoch number was 300. We used a desktop computer with an Nvidia 1080Ti GPU to train the network and achieved 85% accuracy (85% sensitivity and 84% specificity) on the validation dataset. If the likelihood score (generated by the model’s softmax layer) of a case belonging to suspicious class was >0.5, the result would be predicted to be suspicious. Then we converted the TensorFlow model into TensorFlow Lite format using TFLite Converter.^32^ The TensorFlow Lite format is designed to execute models efficiently on devices, which reduces the file size and introduces optimizations that do not affect accuracy.

We have developed an Android smartphone application that implements the MobileNet-based dual-modality OPML and malignancy image classification approach in an easy-to-use format. The application controls the intraoral probe’s camera and LEDs to capture the dual-modality image pair. After the dual-modality oral image pair is obtained, the application can be used to analyze the image pair by our proposed deep learning-based classification method. The Android application uses the TensorFlow Lite Library. The image preprocessing is also implemented on the mobile application, which uses the OpenCV Android library.

We have tested the running speed on three different smartphone CPU and GPU platforms of our proposed method: (1) the Moto G5 smartphone with an Octa-core 1.4 GHz CPU and Adreno 505 GPU, (2) the Samsung Galaxy S7 smartphone with Octa-core (4×2.3  GHz Mongoose and 4×1.6  GHz Cortex-A53) CPU and Mali-T880 MP12 GPU, and (3) the Huawei P30 pro smartphone with Octa-core (2×2.6  GHz Cortex-A76 and 2×1.92  GHz Cortex-A76 and 4×1.8  GHz Cortex-A55) CPU and Mali-G76 MP10 GPU.

## 3 Results

**Fig. 4:**
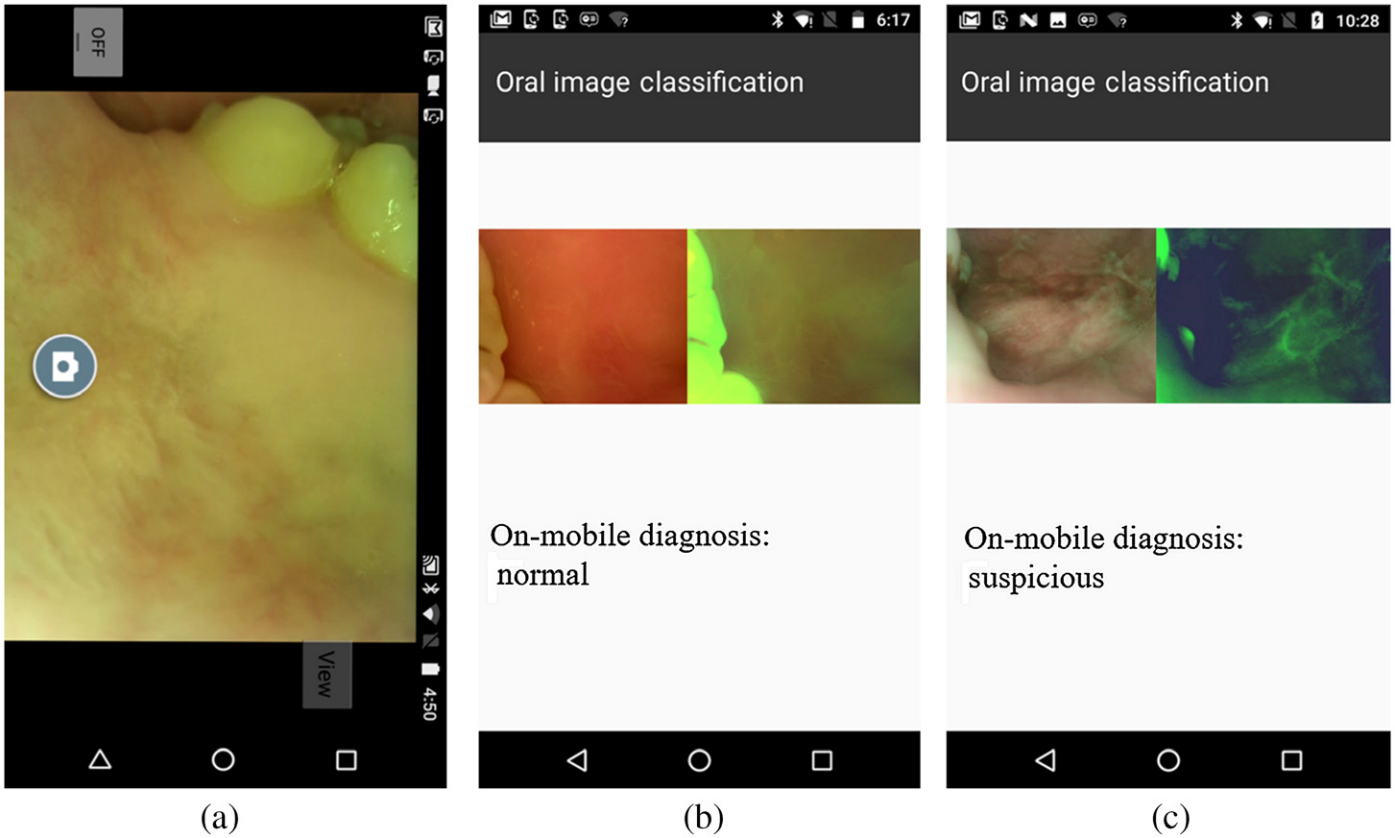
Screenshots of the custom Android application: (a) capture images use our custom application and device; (b), (c) efficient mobile deep learning result.

The finalized lite format MobileNet model is ∼16.3  MB and ideal for smartphone platform operation. Figure 4 shows the screenshots of our customized Android application. It takes ∼300  ms to process one image pair with the Moto G5 Android smartphone.

We have also implemented our proposed dual-modality method with the InceptionV3 network and converted the trained model to the Tensorflow Lite format to run directly on our smartphone platform. The validation accuracy is 85% (85% sensitivity and 85% specificity). The InceptionV3 performance is almost the same as the MobileNet-based approach. However, the model file size is ∼5 times larger (83.3 MB) and 5 times slower running on the Moto G5 smartphone (1981 ms). This experiment shows the MobileNet-based approach can deliver state-of-the-art performance with smaller file size and faster processing speed.

In our experiment, we compared the effect of different optimization algorithms for the dual-modality MobileNet network. To efficiently and effectively train the model to produce accurate results, the optimization strategies and algorithms were used to calculate appropriate and optimum values of the internal parameters of the model. The choice of optimization algorithms for a model plays a significant role in the training process. There are mainly two categories of optimization algorithms: constant learning rate algorithms such as stochastic gradient descent and adaptive learning algorithms such as Adam, Adagrad, and RMSprop. To fairly compare the performances of different optimization algorithms, we kept the learning rate, loss function, and all other settings unchanged. Table 4 shows the RMSProp algorithm having the best performance for this application and the Adadelta optimization algorithm having the longest training time.

**Table 4:** Comparison of validation accuracy and training time by using different optimization algorithms.

Optimization algorithm	Training speed (s)	Performance (validation accuracy) (%)
Adadelta	3771	76.60
Adagrad	3855	81.41
Adam	1867	83.32
GradientDescent	1853	82.63
RMSProp	2057	84.72

**Fig. 5:**
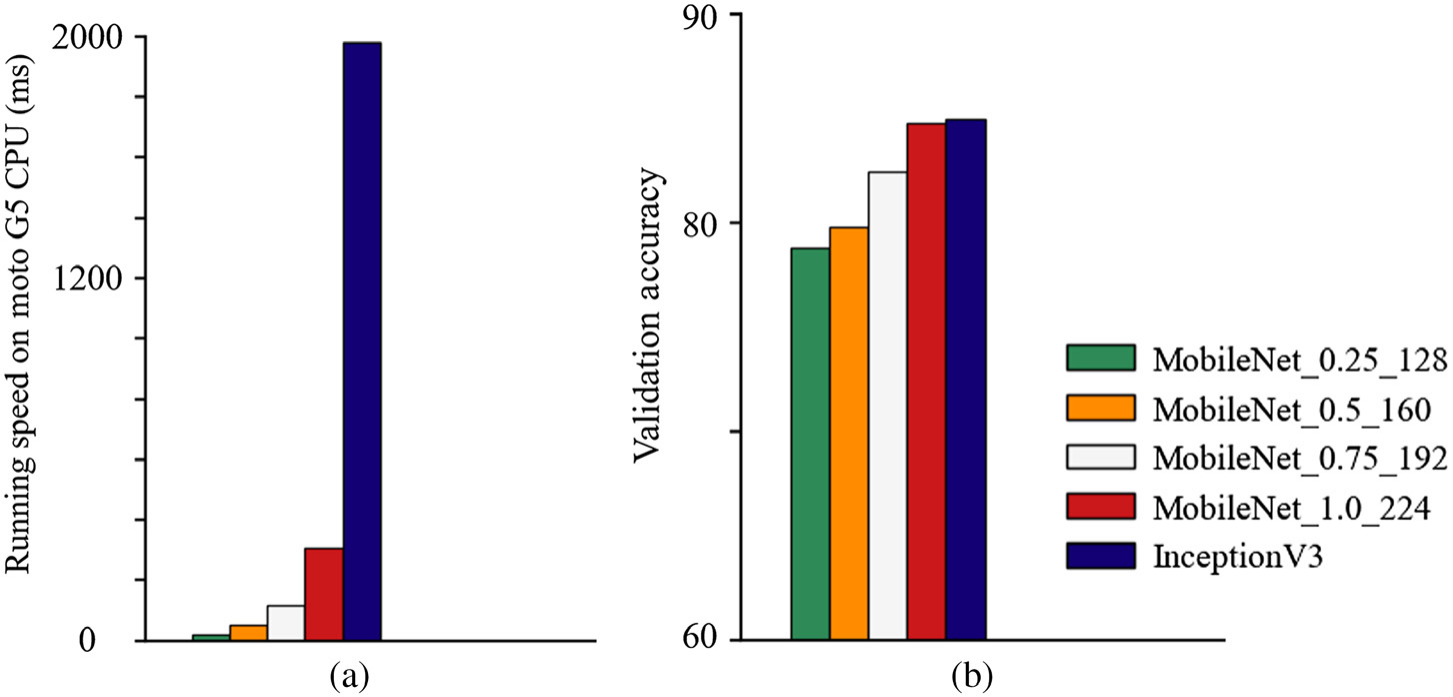
Comparison of running speed on Moto G5 CPU and validation accuracy on our dual-modal oral cancer dataset.

In our experiment, different width multiplier settings of 1, 0.75, 0.5, and 0.25, as well as resolution multiplier settings of 224, 192, 160, and 128 were explored to compare the accuracy, size, and on-device processing time of the model. Figure 5 shows the model with a width multiplier of 1.0 and a resolution multiplier of 224 as having the best accuracy but largest file size and the slowest on-device processing time as compared to other settings (but still smaller and faster than Inception V3). The model with a width multiplier of 0.25 and a resolution multiplier of 128 has the smallest file size and shortest on-device process time but has the worst accuracy performance. Introduction of the hyperparameters that show accuracy trade-offs demonstrate that the speed and file size of the proposed method could be further improved to run faster on more restricted and cost-effective mobile platforms.

**Fig. 6:**
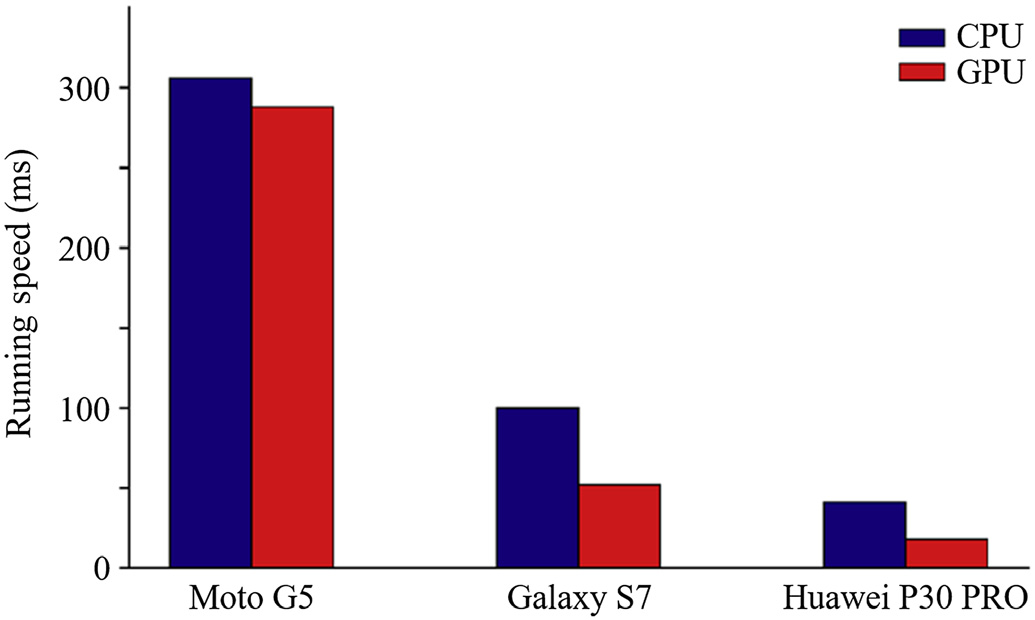
Comparison of running speed (ms per frame) of MobileNet with 1.0 width multiplier and 224 resolution multiplier on different smartphone CPU and GPU platforms.

The comparison of running speeds on three different smartphone CPU and GPU platforms of our proposed method is shown in Fig. 6. The results indicate that the MobileNet-based method could run on different smartphone platforms (different CPUs, different GPUs, and different manufacturers). The runtime is shorter on a more powerful CPU and GPU. However, good performance can still be achieved on a cost-effective smartphone. The results further indicate the running speed is faster on a mobile GPU instead of a mobile CPU.

**Fig. 7:**
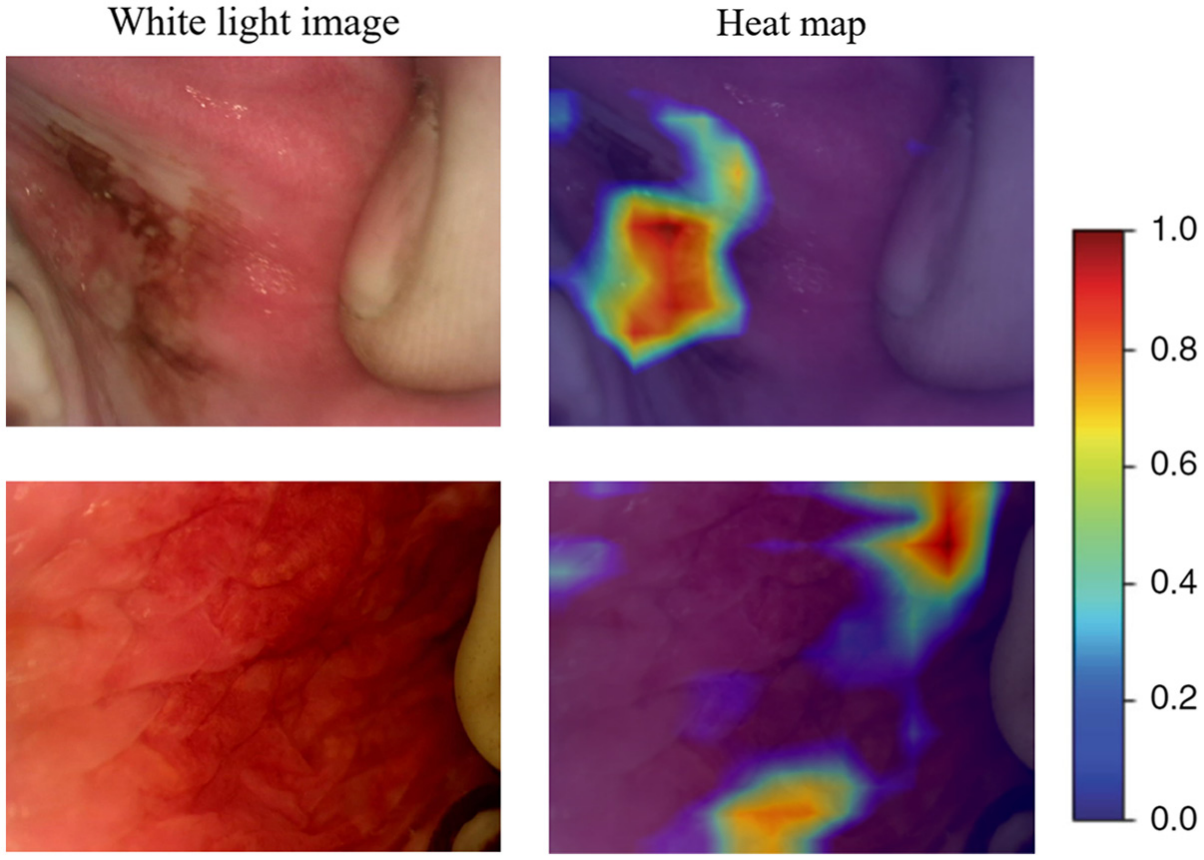
Visualization of two suspicious examples: each example shows the white-light image and corresponding heat map. The red region represents where the neural network is most concentrated.

For investigating the interpretability of the deep learning model, we used gradient-weighted class activation mapping to visualize the network by highlighting the important regions for classification. This helps to visually validate where our network is looking and verify that it looks at the correct patterns in the image and activates around those patterns. It does not represent pixel-wise probability. Examples of two suspicious images are shown in Fig. 7 where the highlighted features in red show where the deep learning model is influenced most. We found that the lesion areas were activated for predicting the result. This demonstrates an encouraging direction for future investigation.

To further challenge and validate the proposed approach, we have tested the proposed methods on the standalone test dataset. Our proposed method achieved 81% accuracy, 79% sensitivity, and 82% specificity on the standalone dataset.

## 4 Conclusion

For oral cancer screening in resource-limited settings, the ideal solution needs to have (1) quick response time, (2) capability to work under restricted conditions such as lack of good medical facilities and a bad internet connection, and (3) high overall performance. Our proposed MobileNet-based dual-modality approach can run quickly with high accuracy on a resource-constrained mobile platform using limited computational power and space. This approach can enable the delivery of deep learning-based oral cancer screening technology into the hands of low-income patients. Our MobileNet-based approach is much more efficient than the conventional deep CNN model. The computational costs and memory requirements are much lower. Our experiment demonstrates the high classification accuracy, fast computing speed, and robustness of the method, which can run on different mobile platforms.

In conclusion, an end-to-end dual-modality deep CNN approach is proposed for automated oral dysplasia and malignancy image classification. This approach classifies dual-modality oral dysplasia and malignancy images fast on mobile platforms. In our preliminary experiments, the approach was able to show accurate results running on different mobile platforms very close to real time. Our method paves the way for oral cancer screening in resource-limited settings. We hope that its power can be further demonstrated and developed for future studies by delivering it to clinicians for use on patients in real time where trained specialists, health facilities, and internet access are limited.
